# Insights into thermal stress effects on performance and behavior of grazing cattle via multimodal sensor monitoring

**DOI:** 10.1038/s41598-025-13264-0

**Published:** 2025-07-31

**Authors:** Regina Eckhardt, Reza Arablouei, Kieren McCosker, Greg Bishop-Hurley, Neil Bagnall, Ben Hayes, Antonio Reverter, Aaron Ingham, Heinz Bernhardt

**Affiliations:** 1 Chair of Agricultural Systems Engineering, TUM School of Life Sciences, Technical University of Munich, 85354 Freising, Germany; 2 Data61, CSIRO, Pullenvale, QLD 4069 Australia; 3 Centre for Animal Science, Queensland Alliance for Agriculture and Food Innovation, The University of Queensland, St Lucia, QLD 4067 Australia; 4 Agriculture and Food, CSIRO, St Lucia, QLD 4067 Australia

**Keywords:** Climate-change impacts, Animal breeding, Data processing

## Abstract

Cattle have been observed to change their behavior and location in response to thermal stress. This study employs a multimodal sensor-based approach to assess if the behavior of grazing cattle changed in response to thermal conditions that occurred during two trials conducted in Queensland, Australia, over late spring and early summer. Each trial involved sixty cattle (Brahman and Droughtmaster) fitted with eGrazor collars containing triaxial accelerometer and GNSS sensors. Cattle were genotyped and weighed weekly, and relevant meteorological data was collected. Accelerometer data was used to classify cattle behavior at five-second intervals into six distinct categories: grazing, walking, ruminating, resting, drinking, and other. GNSS data and satellite imagery were utilized to estimate time spent in open areas, while the Comprehensive Climate Index (CCI) was calculated from meteorological data and used to identify the two warmest and coolest weeks of both trials. Correlation analysis revealed that, during days of higher CCI, cattle increased time in the shade (correlation coefficient $r=0.66$), reduced daytime grazing ($r=-0.49$), shifted grazing to nighttime ($r=0.64$), and prolonged daytime resting ($r=-0.34$). Weather variations had a subtle influence on weight gain, while cattle with increased *Bos indicus* genetic proportion were more active during periods of higher CCI. These findings emphasize the potential of sensor-based monitoring to quantify cattle behavioral responses to variable weather conditions in relevant production environments.

## Introduction

Cattle adapt their behavior to mitigate the impact of thermal stress^1^, adjusting grazing, resting, or shade-seeking patterns in response to temperature fluctuations. For example, cattle often increase shade-seeking behavior or undertake diurnal shifts even under moderate heat conditions to reduce heat stress^2,3^. Previous studies have shown that seasonal variations and forage quality affect grazing behavior in tropical environments^4^, while higher temperatures in temperate climates lead to increased shade-seeking^5^. These findings highlight that cattle modify their behavior in response to temperature changes, even when climate measures remain below thresholds typically associated with heat stress^6^. As climate change drives increasingly variable weather patterns and more frequent extreme events, livestock must adapt their behavior to minimize adverse impacts. Behavioral patterns are key indicators of an animal’s physiological state, with changes providing early signals of compromised well-being^7^. Therefore, understanding how livestock respond to climatic variability is critical for ensuring both animal welfare and sustaining productivity, with potential applications for improving cattle management and sustainability in extreme environments.

Multimodal sensing approaches have proven effective in improving the accuracy of behavioral assessments. For example, Peng et al.^8^ and Arablouei et al.^9^ demonstrated the effectiveness of integrating data from multiple sensor types, specifically accelerometers and Global Navigation Satellite System (GNSS) receivers, to improve animal behavior classification. Previous research has also highlighted the benefits of combining accelerometer sensor data with heat stress measures to offer a more individualized perspective on how animals cope with environmental stressors, for example, through cattle ear tag accelerometers^10^ and rumen boluses^11^.

Building on these insights, this study investigates how livestock, particularly cattle, adapt to varying weather conditions, focusing on the interplay between climatic factors, behavioral responses, and production outcomes through multimodal data. Inspired by Fogarty et al.^12^, in the present study, a multimodal data integration approach is adopted using GNSS data, satellite imagery, accelerometer sensor data, weather data, weight gain measurements, and genotype data:

- *GNSS Data and Satellite Imagery*: The GNSS data, combined with satellite imagery, has been utilized to estimate the duration cattle spend in the open or under the tree canopy, serving as a proxy for shade-seeking behavior. While previous research has utilized direct observations or time-lapse photography to assess shade use by cattle^13–15^, the approach taken in this study utilizes continuous data collected over weeks with minimal observer intervention, enhancing the number of records obtained while preserving key behavioral insights. This is particularly important in extensive systems, where traditional welfare monitoring achieved through direct or recorded observation of individuals is often limited^16^. The inclusion of shade-seeking behavior is particularly relevant, as studies on dairy cows have shown that it plays a critical role in heat stress mitigation, offering relief during warmer conditions^17^. Similarly, Kovacs et al.^18^ have found that animals in shaded areas exhibit fewer lying adjustments, suggesting that being in a shaded area alleviates discomfort and assists cattle to cope with heat stress.
- *Accelerometer Data*: Data collected via accelerometers is an established approach allowing continuous and remote monitoring of animal activity and behavior^9,19–22^. Here, it is included to detect potential behavioral adaptations under varying climatic conditions.
- *Climate Indices from Weather Data*: Climate indices offer valuable insights, but challenges remain in accurately quantifying heat stress and refining measurement techniques^23^, also considering the complex interplay of environmental factors such as temperature, humidity, wind speed, and solar radiation. While commonly used indices such as the Temperature-Humidity Index (THI) are effective in assessing heat stress during hot periods^24^, this study employs the Comprehensive Climate Index (CCI) proposed by Mader et al.^6^, which captures a broader range of environmental conditions, from cold stress to heat stress^25,26^. Notably, cold stress, like heat stress, can significantly affect animal welfare and performance, especially in temperate climates^25,27^.
- *Weight Gain and Genotype Data*: Animal based measures such as weight gain (a key measure of productivity) and genetic composition (quantified as the proportion of Bos indicus genetics in each animal) are examined to provide a comprehensive assessment of climate-driven impacts on livestock performance. Cattle with a greater proportion of Bos indicus genetics are also expected to display enhanced behavioral resilience to heat, maintaining higher activity levels during periods of climatic stress.

We hypothesize that cattle may alter grazing and resting patterns in response to heat stress and further, that this response may be proportional to the level of Bos indicus represented in the genotype. The findings are likely to provide actionable insights for farmers and the livestock industry, informing the development of adaptive strategies in the form of novel breeding traits or management practices that enhance resilience in livestock systems. In particular, it explores the potential to assess the time cattle spend in open areas versus areas covered by tree canopy without direct animal observation. Although the data was not originally collected for thermal stress analysis, this work explores the potential to evaluate the effects of weather on livestock without relying on specialized temperature chambers or controlled diets (i.e., standardized or restricted feeding protocols), approaches typically feasible only for small sample sizes. This may enable large-scale thermal stress assessments without dedicated trial setups, even in non-extreme weather. As climate change alters global weather patterns, understanding how cattle respond to shifting climatic conditions will be crucial for mitigating its impact and ensuring the long-term sustainability of livestock production.

## Methods

### Animal trial and breed composition

This study utilizes accelerometer data collected during two separate cattle experiments. The first experiment was conducted at The University of Queensland’s Queensland Animal Science Precinct (QASP) ($27^{\circ }$32′ 54″ S, $152^{\circ }$20′ 14″ E) in Gatton, QLD, Australia from 21 November to 19 December 2022. The second experiment took place a year later at Darbalara Farm ($27^{\circ }$33′ 21″ S, $152^{\circ }$17′ 23″ E), also in Gatton, QLD, Australia from 21 November to 18 December 2023. Both experiments were approved by the University of Queensland Animal Ethics Committee (approval number 2022/AE000657) and performed in accordance with relevant guidelines and regulations, including the ARRIVE guidelines. A total of 60 Brahman and Droughtmaster cattle, all 15 months old and raised on Gatton Panic, a mixed improved pasture, were used in both studies. The median weight of the animals at the beginning of the trial was 259 kg $( \pm 39 \text { kg})$. Each animal was fitted with an eGrazor collar equipped with a triaxial accelerometer sensor and a GNSS receiver. During the experiments, cattle weight was measured at the start of the trial and weekly thereafter (every Monday) using a static scale.

The Bos indicus genetic content of 48 cattle in the first experiment and 54 cattle in the second experiment was determined as described in Hayes et al.^28^, with values ranging from 47% to 95%. Briefly, the 35k or 50k TropBeef SNP arrays were used to genotype the animals, and GBLUP was utilized to derive Genomic Estimated Breeding Values (GEBV).

### Multimodal analytical framework for sensor data

The first analysis focuses on accelerometer data collected from the collars worn by the animals during the trials. These collars feature embedded systems that log raw accelerometer and GNSS data and perform in-situ recognition of cattle behavior using a pre-trained classification model, as presented by Arablouei et al.^29^. Behavioral predictions can be stored locally or transmitted to a remote server via LoRaWAN or LTE-M^30,31^. The predicted behaviors were aggregated over hourly intervals, allowing for the calculation of time spent exhibiting various behaviors by each animal throughout both trials.

**Fig. 1:**
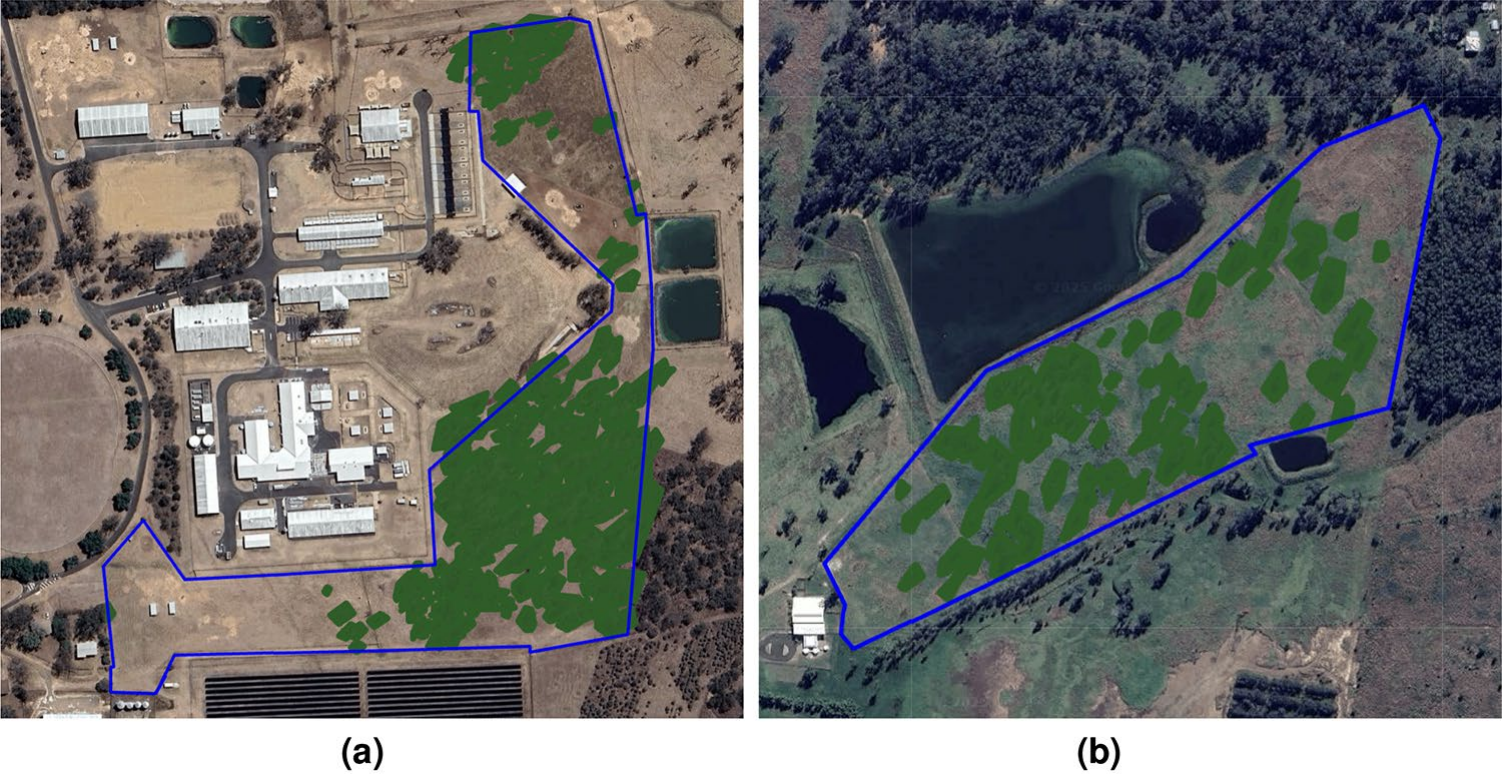
Satellite imagery depicting tree canopy mapping, with the green areas representing tree coverage. Panel (**a**) highlights the Gatton Farm (2022), and panel (**b**) shows the Darbalara Farm (2023). The paddock borders are outlined in blue. Satellite imagery was obtained from Google Earth (web version)^33^, with imagery sources including Maxar Technologies and CNES/Airbus.

The second analysis incorporates GNSS data from the collars. GNSS positions were originally recorded at one-second intervals. The raw GNSS data was aggregated by computing the median longitude and latitude over the same 10-minute intervals, following the approach described by Lund et al.^32^. Satellite imagery of the paddocks was analyzed to map tree canopies, identifying shaded areas by marking the vertices of polygons around tree groups. Due to its public availability and ease of access, the satellite imagery was obtained from Google Earth. While Google does not provide detailed satellite metadata, the images are sourced from providers such as Maxar Technologies and CNES/Airbus, with an estimated spatial resolution of 30–50 cm, depending on the location and zoom level. Tree canopies were mapped through visual interpretation, based primarily on canopy shape and color. A binary variable was added to indicate whether the animal’s median GNSS-derived location during a given interval fell within an open area, from which the hourly time spent in the open was calculated. Figure 1a and b below provide visual representations of the tree canopy mapping for both farms.

Additionally, the distance an animal walked was calculated using the 10-minute GNSS data, applying the Haversine Formula to derive the distances between consecutive positions and then aggregating these values on an hourly basis. The third analysis consists of weather data retrieved from the QASP weather station (Environdata Weather Stations, Australia) at 10-minute intervals. The weather data was used to calculate the CCI^6^, capturing average temperature and other climatic variables relevant to assessing heat stress exposure. In addition to environmental and behavioral data, initial weight, weekly weight gain, and Bos indicus genetic proportion were included in the analysis. Weight gain is a key productivity measure for beef cattle, while genetic proportion provides insights into breed-specific traits. Studies, such as Sprinkle et al.^34^, have highlighted breed-related differences in behavior, particularly in how cattle respond to environmental stress and thermal regulation.

### Data preprocessing

The aggregated behavior predictions, GNSS positions, and weather data were synchronized based on collar IDs and timestamps. Weighing days (Mondays) were excluded from behavioral analysis due to atypical activity patterns, which were induced by the process of mustering and weighing the cattle. The data was further aggregated at a daily level following the methodology outlined in previous related work^35,36^. The maximum daily CCI was used to capture peak thermal stress, while cumulative metrics, such as time spent in the open and distance traveled, were summed across the day. The maximum daily CCI value was retained across all subsets of the data, which were created by dividing the hourly dataframe into two periods - daylight hours and nighttime hours - and then aggregating the data by day. For the daily travel distance, implausible values outside the 0-10 km range were excluded, considering the physical constraints of the enclosed paddocks (measuring on average around 3 hectare).

### Daily ranking for climate exposure

Correlations between weather variables and cattle behavior can often appear weak due to nonlinear relationships and cumulative stress effects. For instance, a single hot day may not significantly influence behavior, whereas consecutive days of high temperatures can lead to notable behavioral shifts^37^.

**Fig. 2:**
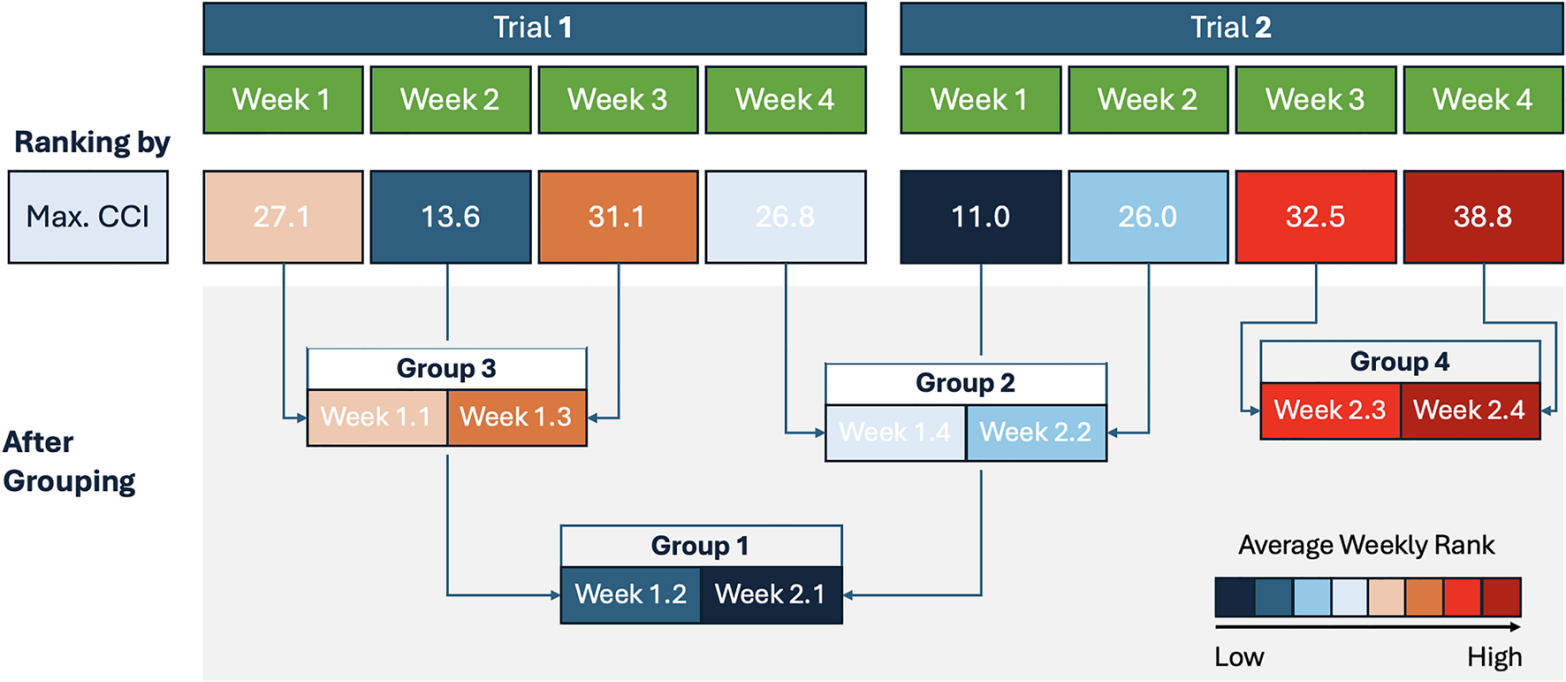
Weekly ranking based on the average maximum CCI across the two trials, with groups formed in ascending order according to their rank.

To address these complexities, we introduce a novel daily ranking approach to enhance correlation analysis. In this approach, we merge data from both trials and rank each day according to its highest daily CCI value starting at rank 1 for the coldest day and assigning higher ranks to progressively warmer days. In this study, daily maximum CCI values ranged from 36 to 52, reflecting moderate to moderately high thermal load conditions typical of subtropical grazing environments. Values around 35–40 are generally considered to represent thermoneutral to mildly warm conditions for Bos indicus cattle, while values above 45 are associated with increasing levels of heat stress, particularly when sustained over multiple days^6^. We then calculated the average rank for each week, providing an estimate of weekly heat exposure. For the correlation analysis, we ranked the weeks based on their average CCI and grouped them into pairs, as shown in Fig. 2. This approach helps account for the cumulative impact of weather, particularly heat stress, which typically arises from consecutive days of high temperatures rather than from a single hot day^38^. Table 1 shows the overall weather statistics for different groups and both trials.

**Table 1:** Weather statistics summarized from daily values for the different groups.

	Temperature ($^{\circ }$C)			Rain (mm)	Wind speed (km/h)	Solar radiation (W/m^2^)
	Max	Min	Mean	Daily Total	Mean	Mean
Group 1	33.0	14.3	23.6	185.2	4.7	291.6
Group 2	33.6	16.5	26.7	101	5.1	441.3
Group 3	37.2	14.1	27.5	1.8	3.9	473.2
Group 4	35.5	18.3	28.9	38	4.9	477.1

### Correlation analysis

Within each group, we computed the Pearson’s correlation coefficient between the CCI and daily behavioral variables, including time spent grazing, resting, ruminating, walking, drinking, time spent in the open, and distance traveled by each animal.

We calculated the correlations separately for daytime and nighttime periods, defined by sunrise at 5 AM and sunset at 6 PM, corresponding to local meteorological data during the trials. This separation aimed to capture potential shifts in behavior towards cooler nighttime hours, as suggested by previous studies^39,40^.

We also computed Spearman’s rank correlation coefficients to verify that our results were not dependent on assumptions of linearity. The outcomes were largely consistent with those obtained using Pearson’s method. Given the improved interpretability of Pearson’s correlation, and considering that the data were cleaned and outliers removed, we chose to report only Pearson’s coefficients in this section.

Weight gain, a key indicator of animal productivity, was also investigated. The relationships between initial weight, weight gain, and behavioral patterns were analyzed to assess whether specific behaviors, influenced by climatic conditions, impacted weight gain in each group. Initial weight was considered to determine whether it affected any of the considered behaviors. Animals had a median weight of 259 kg at the beginning of the trials and reached a median weight of 284 kg by the end of the trials, consistent with expected gains for actively growing steers. The correlation between weekly weight gain and the CCI was calculated to evaluate the effect of temperature on weight gain. Additionally, the correlations between the proportion of Bos indicus genetic content and daily behavior times within each group were examined to test the hypothesis that a higher proportion of Bos indicus reduces sensitivity to higher CCI conditions.

## Results

**Fig. 3:**
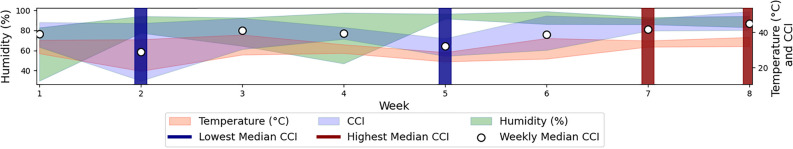
Weather range over time for CCI, temperature, and humidity. Weekly median CCI is shown as white points, with blue and red bars marking the weeks with the highest and lowest CCI, respectively.

The behavioral data analyzed in this study was retrieved from accelerometer sensors embedded in collars during two separate four-week grazing trials conducted in late spring to early summer of 2022 and 2023. Weather data recorded throughout these trials was used to compute weekly mean CCI values. These eight weeks were then ranked and paired based on their CCI values, from the two weeks with the lowest to the two weeks with the highest CCI values. A summary of the weather conditions and the median values of time spent on various behaviors during each week is provided in Fig. 3 and Table 2, respectively.

**Table 2:** Summary of the median daily time (in hours) spent on the considered behaviors across the eight weeks of both trials. Values represent median ± median absolute deviation (MAD).

	Week 1	Week 2	Week 3	Week 4	Week 5	Week 6	Week 7	Week 8
Time in Sun (h)	10.7 ± 0.8	8.3 ± 1.0	12.2 ± 0.7	10.2 ± 1.2	10.6 ± 0.9	10.3 ± 1.3	10.2 ± 0.8	8.5 ± 1.7
Grazing (h)	6.7 ± 0.7	6.6 ± 0.8	6.4 ± 0.5	7.5 ± 0.7	6.8 ± 0.7	7.8 ± 0.7	6.7 ± 0.9	7.0 ± 1.1
Ruminating (h)	2.8 ± 0.4	2.8 ± 0.4	2.9 ± 0.4	2.2 ± 0.4	3.4 ± 0.3	2.6 ± 0.3	3.6 ± 0.5	3.4 ± 0.4
Resting (h)	2.5 ± 0.4	2.2 ± 0.5	2.6 ± 0.4	2.1 ± 0.5	1.9 ± 0.4	1.6 ± 0.5	1.5 ± 0.4	1.4 ± 0.5
Walking (h)	0.6 ± 0.2	0.8 ± 0.2	0.7 ± 0.2	0.9 ± 0.2	0.6 ± 0.2	0.6 ± 0.2	0.8 ± 0.2	0.8 ± 0.2
Drinking (h)	0.1 ± 0.1	0.1 ± 0.0	0.1 ± 0.1	0.1 ± 0.1	0.0 ± 0.1	0.0 ± 0.0	0.1 ± 0.1	0.1 ± 0.1
Other (h)	0.1 ± 0.1	0.1 ± 0.1	0.2 ± 0.1	0.1 ± 0.1	0.1 ± 0.1	0.1 ± 0.1	0.2 ± 0.1	0.2 ± 0.1
Distance walked (km)	2.5 ± 0.2	3.5 ± 0.6	3.2 ± 0.4	3.9 ± 0.6	2.9 ± 0.4	3.1 ± 0.7	2.9 ± 0.4	3.2 ± 0.4

### High CCI conditions

**Fig. 4:**
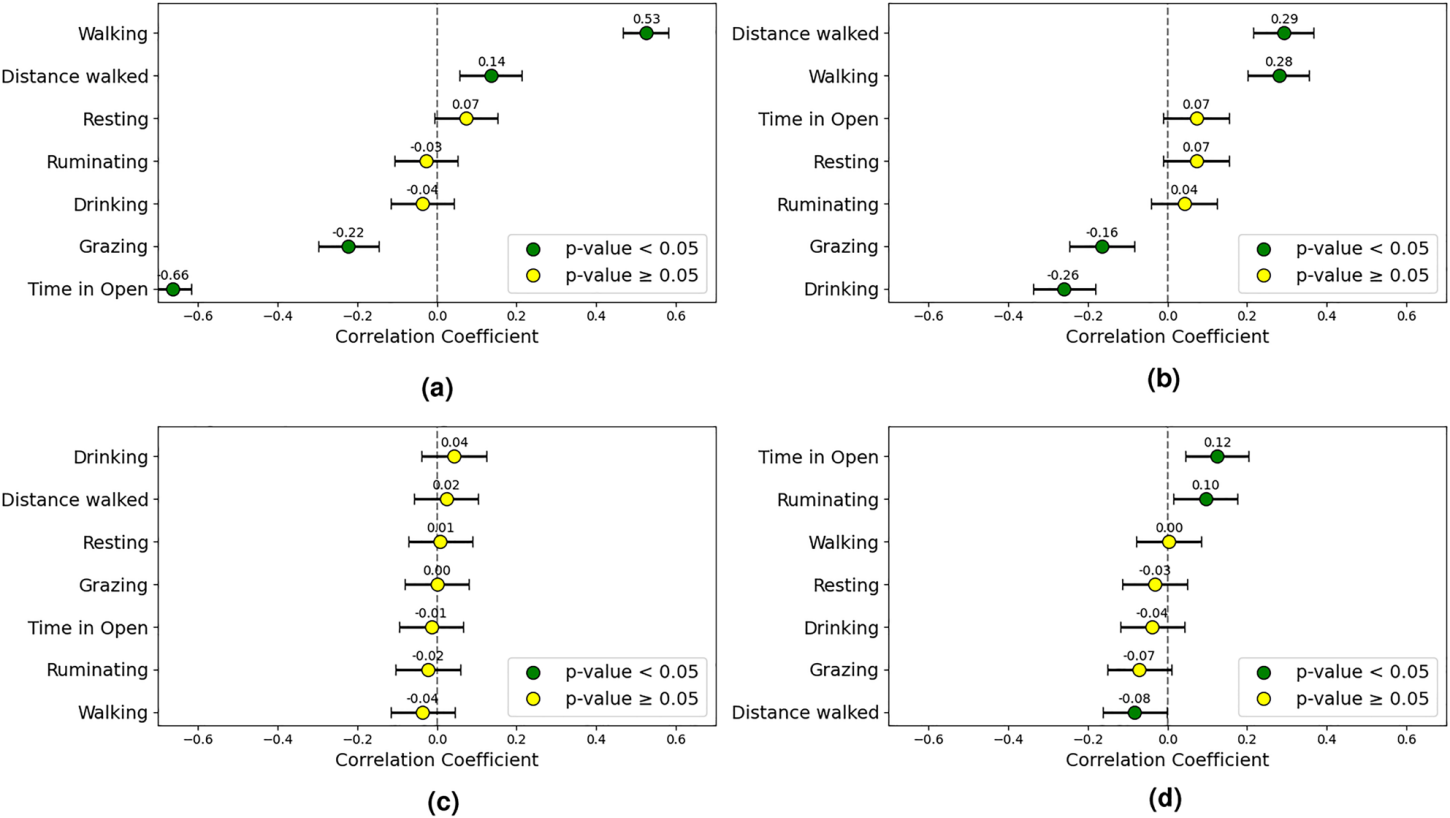
Correlation plots including (**a**) CCI, (**b**) Bos indicus content, (**c**) initial weight, and (**d**) weekly weight gain, examining the relationship between animal behavior and these factors over the full 24-hour period in high CCI conditions. The correlation coefficients and their 95% confidence intervals are displayed.

During the two warmest weeks of both trials correlations among the CCI, cattle behaviors, and weight gain were analyzed for full-day, daylight, and nighttime periods. Since “time spent in the open” is used here to assess shade seeking behavior, it is only relevant to the daylight period, and was therefore excluded from full-day analyses. Higher CCI conditions were associated with increased walking (0.53) and reduced grazing (-0.22). The proportion of Bos indicus genetic content correlated positively with walking (0.28) and negatively with grazing (-0.16) and drinking (-0.26) behaviors. Weight gain was weakly correlated with ruminating (0.10). The full-day correlation results are shown in the forest plots of Fig. 4.

#### Daytime behavior correlations

Time spent in open areas negatively correlated with CCI (-0.66) and grazing time decreased (-0.49), while resting (0.34) and ruminating (0.38) increased with rising CCI. Walking behavior showed a positive correlation (0.36) with CCI.

Minor correlations between the Bos indicus proportion and different behaviors were observed. These included positive correlations with ruminating (0.14) and walking (0.30) behaviors as well as distance walked (0.29), and negative correlations with grazing (-0.15) and drinking (-0.29) behaviors.

**Fig. 5:**
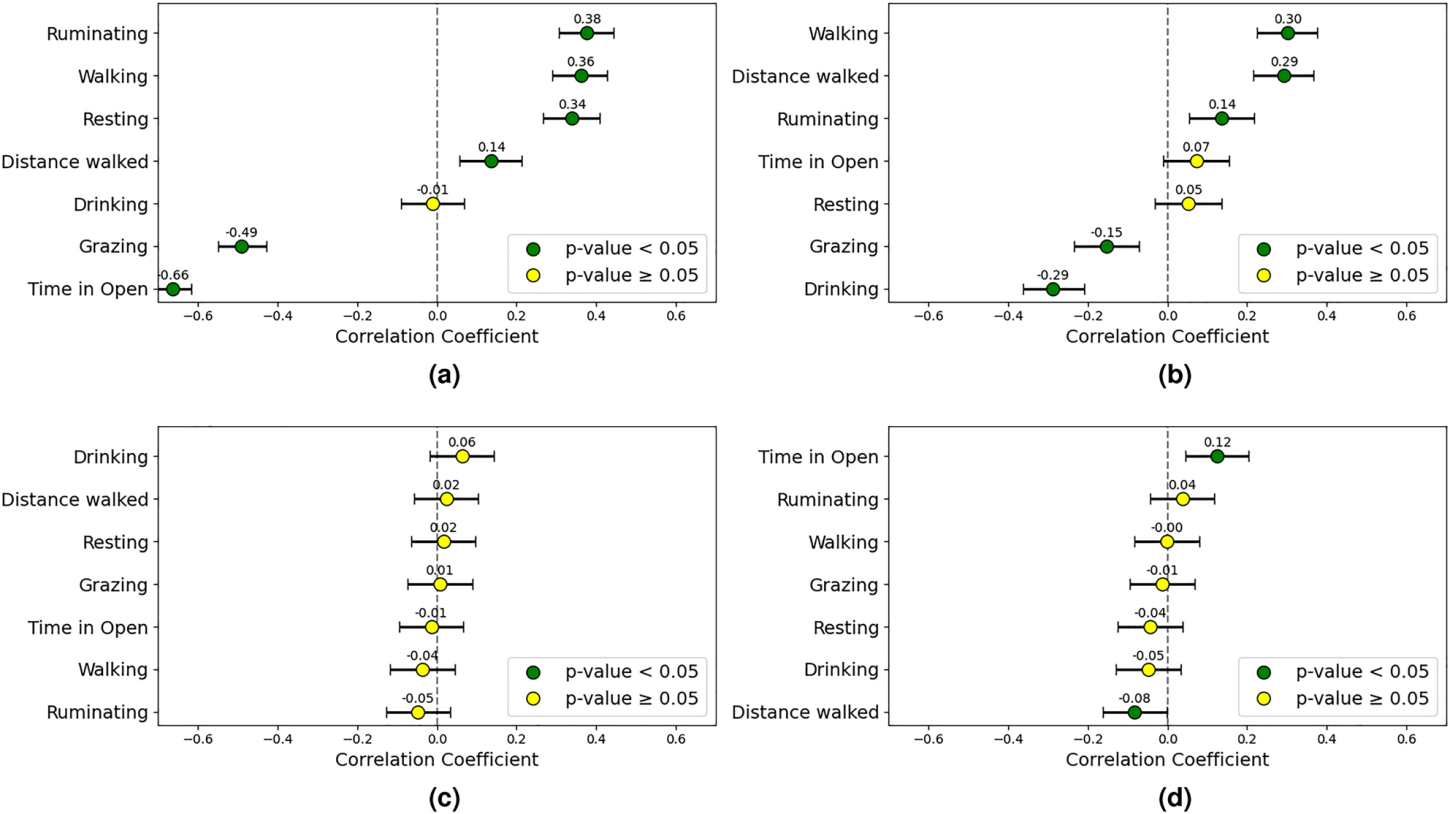
Correlation plots including (**a**) CCI, (**b**) Bos indicus, (**c**) initial weight, and (**d**) weekly weight gain, examining the relationship between animal behavior and these factors during daylight hours in high CCI conditions. The correlation coefficients and their 95% confidence intervals are displayed.

Interestingly, while cattle spend less time in the open during the warmest weeks, there was a positive correlation between time spent in the open and weekly weight gain (0.12). No significant correlation was found between initial weight and daily behavior times in high CCI conditions. Figure 5 provides an overview of daytime behavior correlations during the two warmest weeks of both trials.

#### Nighttime behavior correlations

A strong positive correlation between the CCI and both grazing (0.64) and walking (0.63) times was found during nighttime hours. In contrast, ruminating (-0.41) and resting (-0.29) times decreased with increasing CCI.

Minor correlations were observed between the Bos indicus proportion and nighttime behaviors, including a positive correlation with walking time (0.16) and distance walked (0.29), and a negative correlation with drinking time (-0.15).

**Fig. 6:**
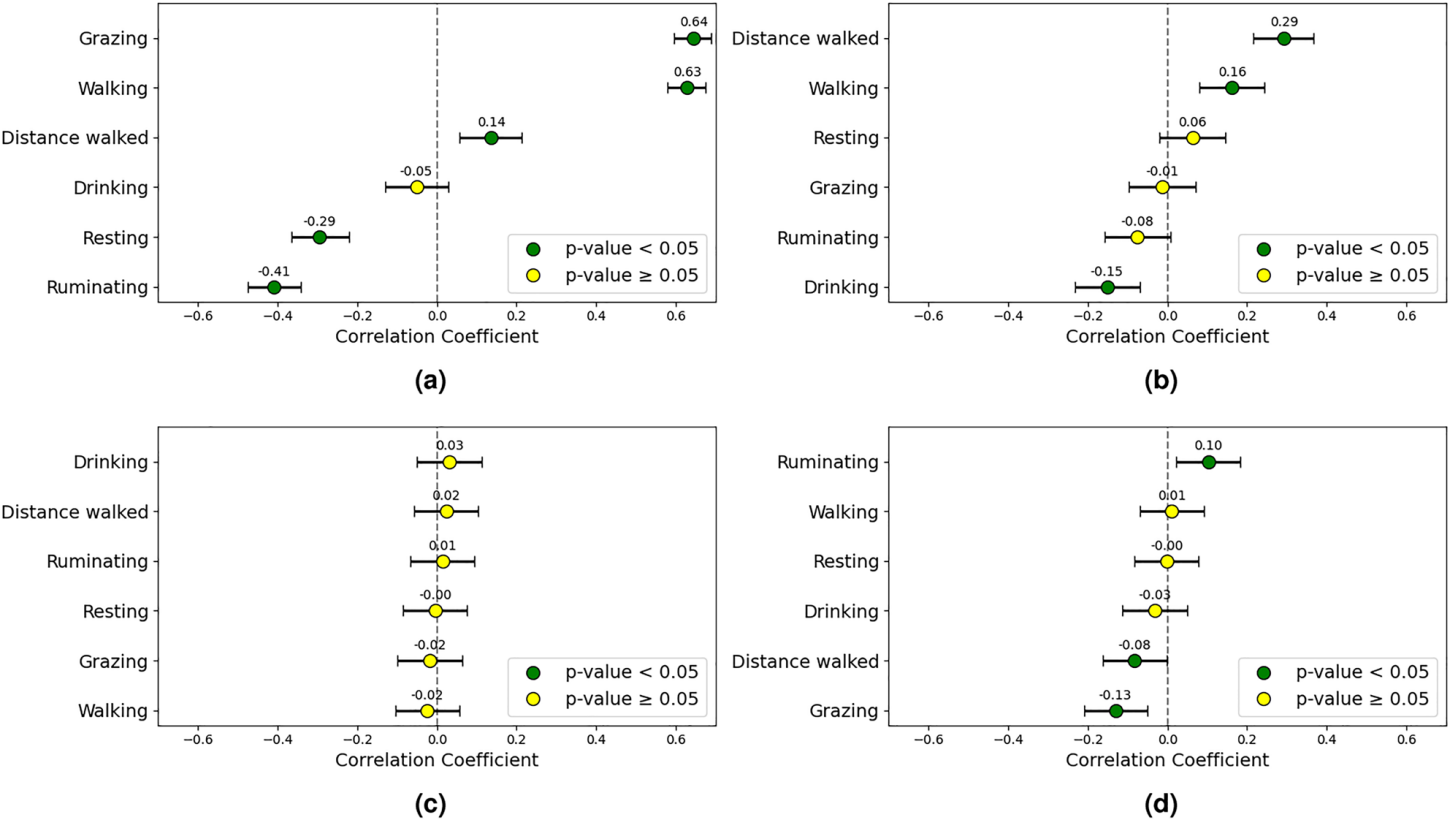
Correlation plots including (**a**) CCI, (**b**) Bos indicus proportion, (**c**) initial weight, and (**d**) weekly weight gain, examining the relationship between animal behavior and these factors during nighttime hours in high CCI conditions. The correlation coefficients and their 95% confidence intervals are displayed.

Moreover, there was a slight negative correlation between nighttime grazing and weight gain (-0.13), as well as a weak positive correlation between nighttime ruminating and weight gain (0.10). Other behaviors showed minimal influence on weight gain, and no significant correlations were found with initial weight, as shown in Fig. 6.

### Low CCI conditions

**Fig. 7:**
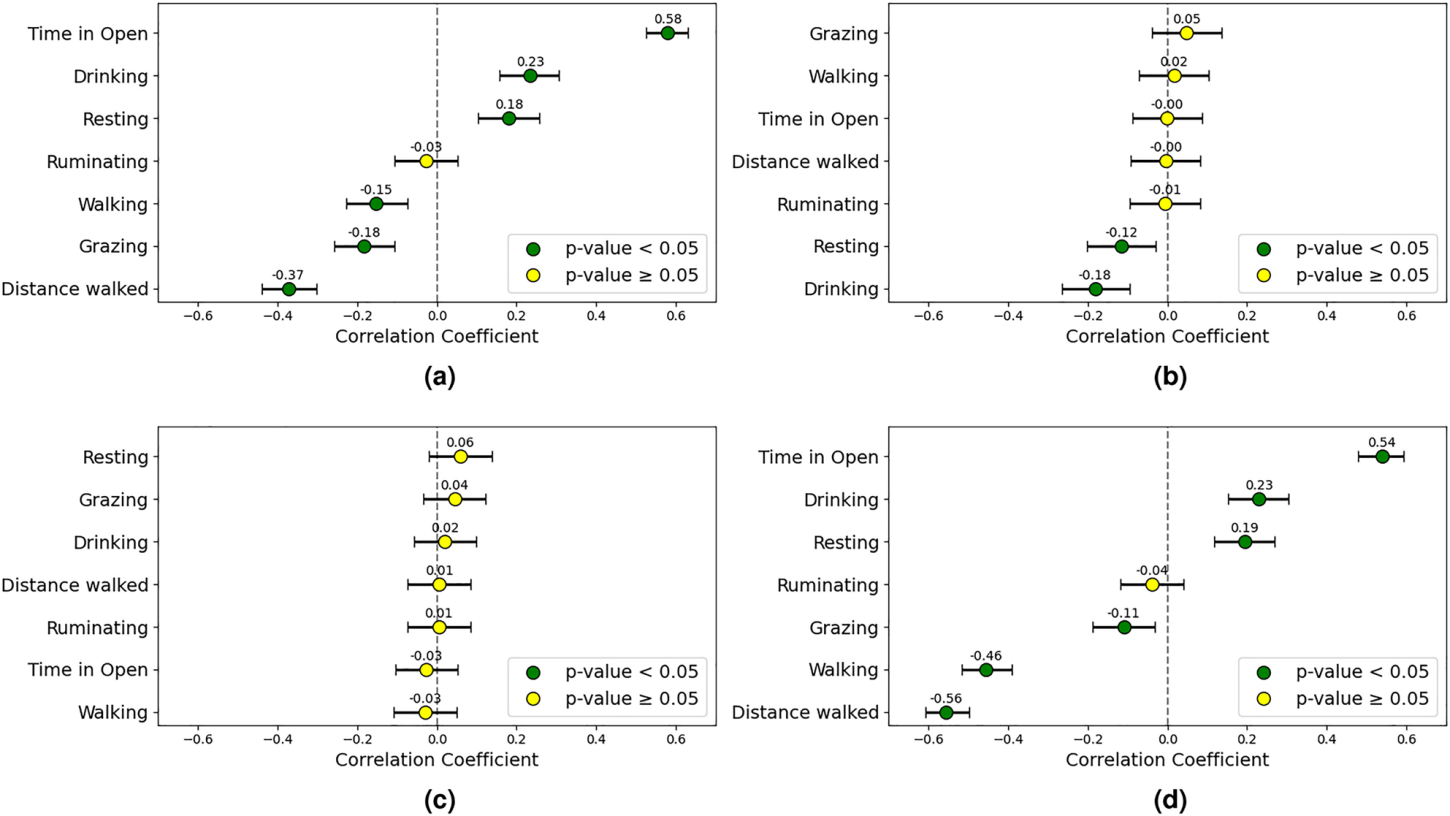
Correlation plots including (**a**) CCI, (**b**) Bos indicus proportion, (**c**) initial weight, and (**d**) weekly weight gain, examining the relationship between animal behavior and these factors over the full 24-hour period in lower CCI conditions. The correlation coefficients and their 95% confidence intervals are displayed.

During the two coolest weeks of both trials, the relationship between the CCI and cattle behaviors across full-day, daylight, and nighttime hours was examined, alongside the correlations with weight gain. Under lower CCI conditions, cattle exhibited increased drinking and resting activity, while grazing and walking behavior times were reduced. The proportion of Bos indicus showed a weak negative correlation with resting (-0.12) and drinking (-0.18) behaviors. Weight gain was positively correlated with drinking (0.23) and resting (0.19) behaviors, and negatively correlated with grazing (-0.11) and walking (-0.46) behaviors. The full-day results are presented in the forest plots in Fig. 7.

#### Daytime behavior correlations

A positive correlation was observed between time spent in open areas and CCI (0.58). Additionally, a negative correlation was found between the CCI and grazing time (-0.28). Instead, animals increased their resting (0.32) and drinking (0.34) times under milder conditions. Interestingly, there was a negative correlation between distance walked and the CCI in cooler weather (-0.37).

**Fig. 8:**
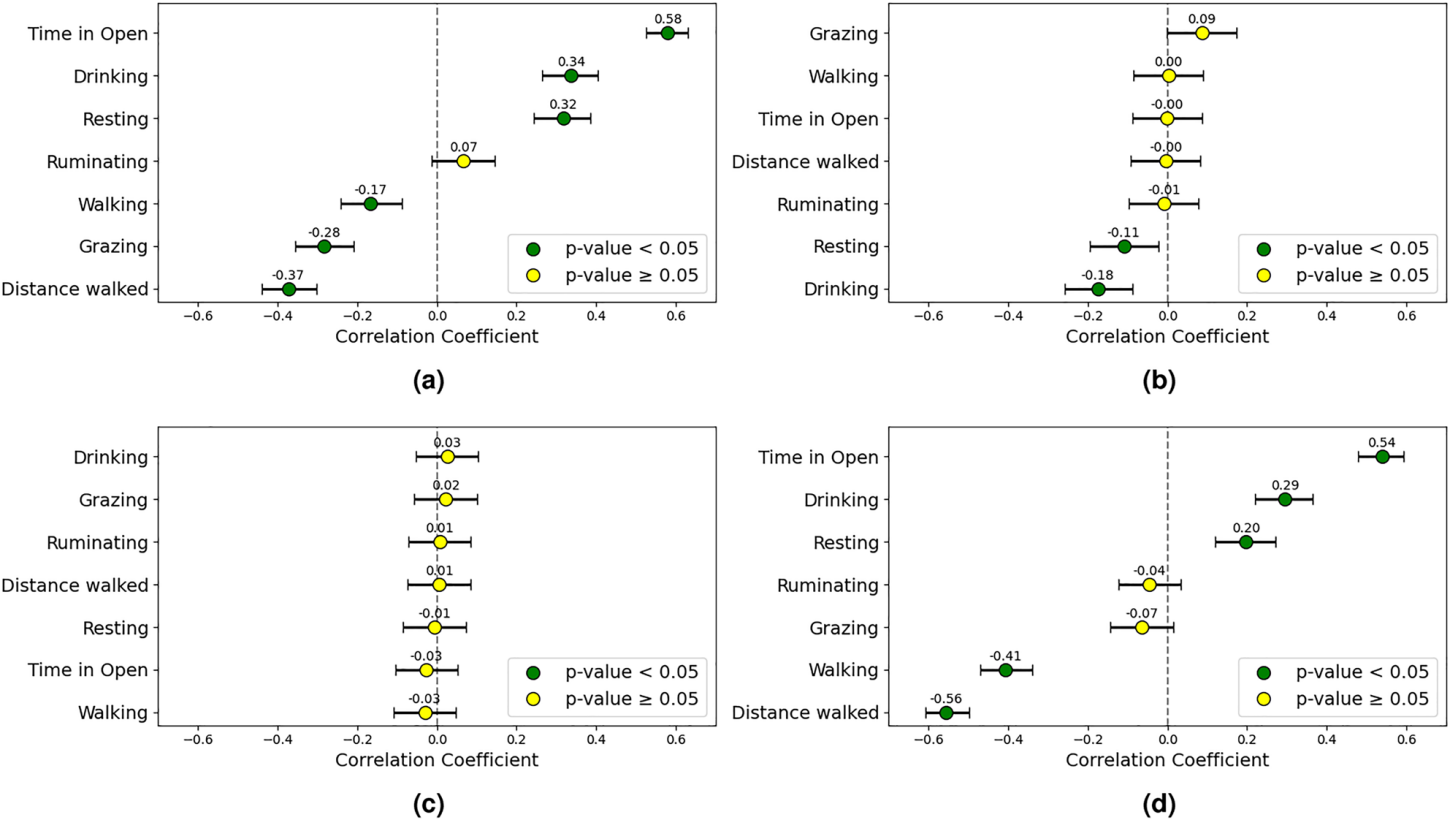
Correlation plots including (**a**) CCI, (**b**) Bos indicus, (**c**) initial weight, and (**d**) weekly weight gain, examining the relationship between animal behavior and these factors during daylight hours in lower CCI conditions. The correlation coefficients and their 95% confidence intervals are displayed.

The Bos indicus proportion was negatively correlated with drinking (-0.18) and resting (-0.11) times. Weight gain was positively correlated with time spent in the open (0.54) as well as drinking (0.29) and resting (0.20) times, while showing negative correlations with walking time (-0.41) and distance walked (-0.56). Initial body weight showed no significant correlation with the considered behaviors. A summary of these correlations is presented in Fig. 8.

#### Nighttime behavior correlations

During cooler periods, a slight positive correlation was observed between CCI and grazing time (0.08). In contrast, time spent ruminating (-0.12) and distance walked (-0.37) exhibited negative correlations with CCI. Bos indicus proportion showed a weak negative correlation with drinking time (-0.12).

**Fig. 9:**
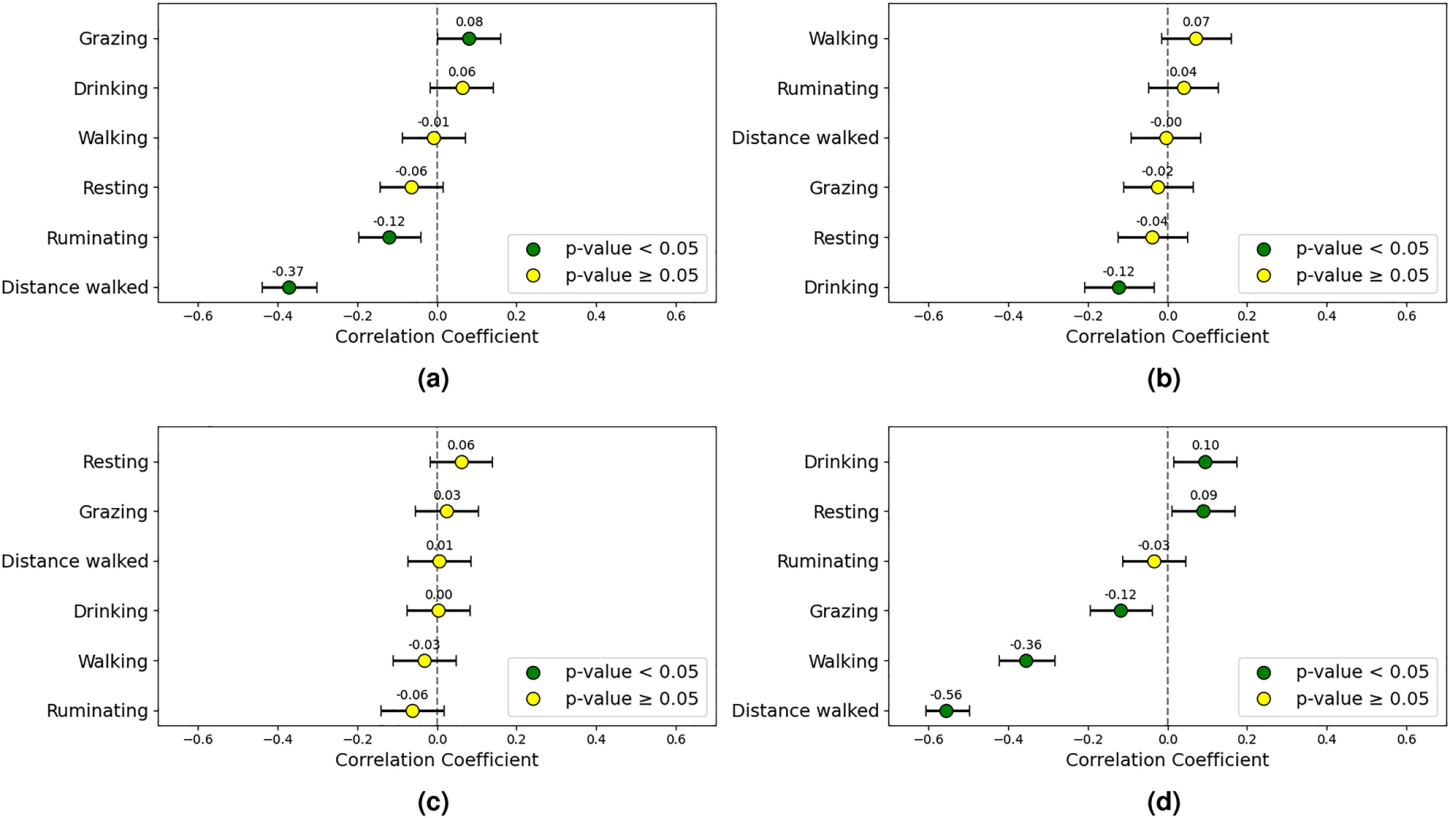
Correlation plots including (**a**) CCI, (**b**) Bos indicus, (**c**) initial weight, and (**d**) weekly weight gain, examining the relationship between animal behavior and these factors during nighttime hours in lower CCI conditions. The correlation coefficients and their 95% confidence intervals are displayed.

Regarding weekly weight gain, notable negative correlations emerged with distance walked (-0.56) and walking time (-0.36), and a slight negative correlation with grazing time (-0.12), suggesting that increased movement at night may hinder weight gain. Weak positive correlations with drinking (0.10) times were observed. The findings underscore the complexity inherent in the interactions between nighttime activity and weight gain under cooler conditions. Initial weight did not significantly correlate with any of the measured behaviors. Figure 9 presents a summary of nighttime behavioral correlations observed during the two coolest weeks of the trials.

### Correlation of weight gain with CCI and Bos indicus proportion

Under high CCI conditions, a weak negative correlation (-0.10) was observed between CCI and weight gain, suggesting that prolonged periods of elevated temperatures may slightly inhibit weight gain.

**Fig. 10:**
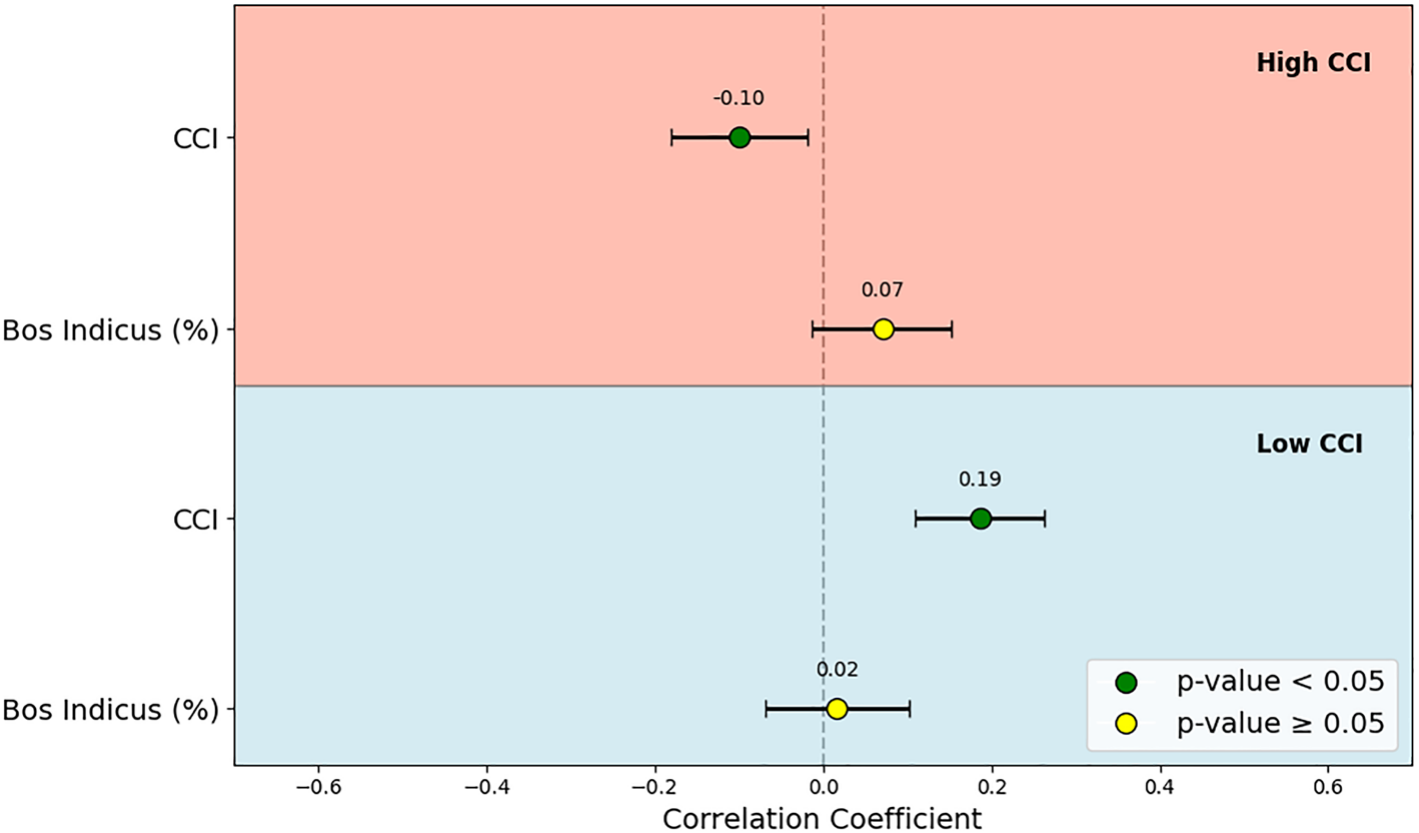
Correlation plots for weekly weight gain, examining the relationship between CCI as well as the Bos indicus proportion and these factors during nighttime hours in both high and low CCI conditions. The correlation coefficients and their 95% confidence intervals are displayed.

In contrast, under low CCI conditions, a slight positive correlation (0.19) was observed between CCI and weight gain, suggesting that cooler temperatures may marginally enhance weight gain. However, no significant correlation was found between the Bos indicus proportion and weight gain under these cooler conditions, indicating that breed composition had minimal influence on weight gain during periods of lower thermal stress. The correlation coefficients for weight gain, with the CCI and the Bos indicus proportion under both high and low CCI conditions are summarized in Fig. 10.

## Discussion

The correlation analysis across both high and low CCI conditions offers valuable insights into how cattle adjust their behavior in response to temperature fluctuations, with only modest effects observed on weight gain, which is expected given the short study duration. In higher CCI conditions, cattle exhibited behavioral shifts through increased shade-seeking behavior, reduced time spent in open areas, and decreased grazing activity. Instead, they devoted more time to ruminating and resting. Although rumination is energetically costly and can increase body temperature, this shift may reflect a behavioral choice by the cattle to prioritize activities that minimize heat exposure. When faced with the option of grazing in the sun or ruminating in the shade, cattle may opt for the latter to avoid the more extreme heat-generating activities associated with grazing^41^.

Interestingly, while cattle spent less time in the open during the high CCI weeks, there was still a positive, albeit weak, correlation between time in the open and weight gain. This is consistent with prior research on dairy cattle^42,43^, which found that increased sunlight exposure contributes to improved weight gain, potentially through both increased feed intake and hormonal changes associated with prolonged sunlight exposure. However, in this study, the observed correlation may be more likely related to feed availability than sunlight exposure, as forage is usually more abundant and accessible in open areas. Cattle that spent more time in these areas may therefore have had increased access to forage, contributing to their higher weight gain. This interpretation is consistent with findings of dos Santos et al.^44^, who demonstrate that increased forage availability and nutritional value can lead to enhanced cattle performance and weight gain. The observation that cattle alter the amount of time spent in direct sunlight in response to thermal conditions may provide the basis of a novel thermal resilience trait that measures the duration of time cattle spend grazing in open areas despite increased heat exposure. Such behaviors may reflect an ability to maintain feeding activity under heat stress, which could have important implications for livestock productivity. This interpretation is supported by findings that feed intake typically declines under heat stress conditions^45^, but can be maintained through environmental mitigation strategies such as shade or cooling^46^.

Although nighttime grazing activity increased under high CCI conditions, this shift may reflect a behavioral adaptation to thermal stress rather than a strategy to enhance intake. The observed weight gain patterns indicate that cattle were substituting daytime grazing with nighttime activity to avoid heat exposure, rather than increasing their overall foraging effort. A weak negative correlation between heat exposure (measured by the number of consecutive high CCI days) and weight gain suggests that higher temperatures may slightly impede weight gain, likely due to altered grazing patterns driven by heat stress. While cattle generally remain active at night, research indicates that they typically graze less during nighttime compared to daytime^47,48^ and the data revealed that total daily grazing time did not significantly increase, suggesting that nighttime grazing, while potentially beneficial for maintaining nutritional intake^49^, did not lead to a net improvement in weight gain. Moreover, although cattle did engage in nighttime grazing, the effect on intake volume remains uncertain. Limited visibility during nocturnal hours may restrict the ability to identify pasture height, availability and quality, potentially leading to reduced intake compared to daytime grazing. This aspect warrants further investigation in future studies. Additionally, under extreme weather conditions where daytime grazing becomes harder for animals, a complete shift to nighttime grazing is unlikely to be a viable solution, as nocturnal intake alone has previously been shown to be insufficient to sustain optimal weight gain^50^.

In contrast, cooler weather conditions resulted in different behavioral responses. Cattle increased daytime grazing activity, likely taking advantage of more favorable temperatures for foraging. This increase in active foraging was accompanied by reduced time spent ruminating and resting during the day. Interestingly, cattle walked less as temperatures increased slightly, suggesting that they adjusted their activity patterns in response to the more favorable conditions. These behavioral changes may reflect cattle’s response to brief, unexpected warm spells in typically cooler environments, which could induce heat stress, even if the temperatures do not reach hazardous levels.

At night, distinct behavioral differences were observed between high and low CCI conditions. While nighttime grazing activity increased in both scenarios (likely as a response to daytime heat stress), this did not appear to lead to a corresponding increase in weight gain. It remains unclear whether the shift to nocturnal grazing influences overall intake levels and thereby affects growth performance. In cooler conditions, cattle spent more time ruminating and resting at night. However, this behavioral shift did not result in significant weight gain, reinforcing the notion that factors such as feed availability and overall energy expenditure likely play a greater role in influencing weight gain than nocturnal rest and rumination. Additionally, the proportion of Bos indicus genetic content within the herd did not appear to have a strong influence on behavioral responses to temperature fluctuations. Minor correlations were observed, including increased walking in high CCI conditions and more frequent rumination in cooler conditions, but these effects were not statistically significant. Nevertheless, these subtle trends merit further investigation with larger sample sizes to explore potential breed-specific adaptations to environmental stressors.

Future research can further explore the physiological and behavioral mechanisms that underlie thermal resilience in grazing cattle. Investigating traits that enable certain individuals to maintain feeding activity and productivity under heat stress may inform breeding strategies aimed at enhancing climate adaptation. Additionally, integrating precision tools such as high-resolution aerial mapping and activity tracking can improve assessments of environmental exposure and behavioral responses, supporting more targeted management interventions. Future research can also extend monitoring durations to capture long-term behavioral responses to climatic stress, refining models for optimizing cattle productivity in variable environments. Additionally, incorporating physiological metrics such as heart rate and rumen temperature can provide a deeper understanding of cattle stress responses. Advancements in machine learning can further enhance behavior classification, improving predictive capabilities for livestock management. In particular, generative artificial intelligence (AI) techniques offer the potential to process large-scale sensor data efficiently, enabling the development of informative embeddings that better capture behavioral adaptations. These learned representations can be leveraged to refine predictive models, enhancing the ability to anticipate stress responses and optimize cattle management strategies. Expanding this research to diverse climatic regions and pasture conditions will further strengthen the generalizability of findings, contributing to the development of robust climate adaptation frameworks for sustainable livestock agriculture.

This study characterizes the response of Brahman-based cattle in subtropical conditions across a daily maximum CCI range of 36-52. While correlations were generally modest - reflecting the focused and realistic weather range - a notable strength of this design is that it can reveal meaningful relationships between environmental conditions and cattle behavior without relying on extreme heat stress scenarios. This underscores the potential to understand climate-behavior dynamics under typical weather conditions, thereby making the findings more broadly applicable to real-world production systems. It is important to acknowledge certain methodological considerations. The mapping of tree canopies, based on satellite imagery from Google Earth, may not accurately reflect actual shade availability during the study period. Changes in tree canopy structure over time and variations in sun angle likely influenced shade coverage. Future research can improve accuracy by incorporating high-resolution aerial imagery (e.g., drone-based mapping) during the trial period, allowing for a more precise assessment of canopy coverage and sun exposure. Additionally, considering the animals’ body size relative to available shade can help determine whether certain individuals experienced inadequate access to shade or shelter.

This study highlights the behavioral adaptations of cattle in response to temperature fluctuations, demonstrating that even modest climatic changes can influence grazing patterns, resting behavior, and weight gain. Although cattle in high CCI conditions shifted grazing to cooler nighttime hours, this did not significantly improve weight gain, suggesting that other factors, such as feed intake and metabolic efficiency, play a more prominent role. In contrast, increased daytime grazing in cooler conditions was associated with improved weight gain, emphasizing the importance of managing environmental factors to optimize cattle productivity. The weak correlations between behavior and weight gain further suggest that resilience traits, feed availability, and pasture quality should be considered in future research. This study offers valuable insights into how cattle management strategies can be adapted to mitigate the effects of climate variability, supporting efforts to enhance livestock health, welfare, and productivity.

## Conclusion

This study highlights the role of climatic variability in shaping cattle behavior and performance, particularly weight gain. The findings reveal that cattle modify their activity patterns in response to temperature fluctuations, exhibiting heightened shade-seeking behavior during hotter periods and increased grazing during cooler periods. While correlations between weight gain and climate variables were weak, environmental conditions clearly influence behavioral adaptations. Consistent with our hypotheses, we found that both heat stress (as captured by CCI) and genetic composition (Bos indicus proportion) were associated with distinct patterns in cattle behavior. By employing a multimodal approach, this study underscores the value of integrating sensor-based data in livestock monitoring, providing insights into climate adaptation strategies, such as providing the basis for developing novel traits for breeding programs or in validating the importance of maintaining a shade canopy in the grazing environment. These findings contribute to a better understanding of how cattle behavior and productivity respond to climatic variability, informing future efforts to enhance livestock resilience and management under changing environmental conditions.

## Data Availability

Data can be made available upon request from the corresponding author at regina.eckhardt@tum.de.
